# Doing neuroscience in Brazil: The best Brazilian theses in Neuroscience 2005-2007 III Brazilian Congress on Brain, Behavior and Emotions

**Published:** 2007

The theses presented in post-graduate programs across Brazil between 2005-2007 in the area of Neurosciences, and submitted to the III Brazilian Congress on Brain, Behavior and Emotions to be held in Bento Gonçalves, Rio Grande do Sul on April 19-22, 2007, have been evaluated by a special committee headed by Prof. Jair Mari and Prof. Jaderson Costa da Costa. The abstracts of the best theses are now presented here.

## ABSTRACT-1

**INFERENCE PROCESS OF COMPLEX HUMAN TRAJECTORIES REQUIRES INTERNAL MODELS OF ACTION: BEHAVIOURAL EVIDENCE**

**G Saunier^1,2^,T Pozzo^2^, CD Vargas^1^**

^1,2^Laboratório de Neurobiologia II, Instituto de Biofísica Carlos Chagas Filho, Universidade Federal de Rio de Janeiro, Rio de Janeiro, Brasil. ^1^INSERM-ERM 0207 Motricité-Plasticité, Université de Bourgogne, Campus Universitaire, BP 27877, F-21078 Dijon, France. E-mail: ghis@biof.ufrj.br

### Objectives:

How do we visually extrapolate the final position of a biological motion like for instance the final position of a hand that reaches an object located behind a wall? What is the human ability to reconstruct the hidden part of a moving target? A previous study of our group demonstrated that the human capacity to infer about the final position of simple vertical arm motion relies on an internal model of our motor knowledge. The purpose of this report was to verify if the use of internal models of action during inference process can be generalized for intransitive and complex human motions like Sit to Stand (STS) or Back to Sit (BTS).

### Methods:

11 healthy subjects (5 men and 6 women, 27±8 years old) participated in this experiment. The study consisted in estimating the final position of STS or BTS shoulder’s trajectories, according to a biological or non biological kinematics. Four kinds of movement were displayed. Two of movements consisted in the dot motion on the screen depicting shoulder STS or BTS trajectories as recorded from subjects’ movement (we call these motions biological, since kinematics corresponds to movements with well-known motor laws). For the two other movements, a conflict was introduced on shoulder velocity profiles. Specifically, the STS motion of the dot was displayed according to the velocity profile corresponding to a BTS motion (violation of the biological motion, STS N) and the BTS motion of the dot was displayed according to STS velocity profile (BTS N). A unique light dot represented this displacement. The last part of the trajectory (i.e. 35%) was occulted. Subject gave its response by using the mouse. We measured the constant error (i.e. the difference between actual and true position) and variable error (the standard deviation for each condition) in mm.

### Results:

An ANOVA test showed a significant motion effect (F(1.10)=25.1, p=0.0005). Scheffe’s post hoc analysis revealed that the precision was greater for biological motion (12.3±2.5mm) than non biological motion (15.5±2.4mm).

### Conclusion:

The effect of velocity profile suggests the participation of internal representations to infer the final part of intransitive complex motion. We hypothesize that in the absence of visual input, a forward model would contribute to estimate target motion in time and space by converting the motor planning into a predicted visual representation. Thus, the motor laws governing the production of this complex movement must be implied during task performance.

***Financial support:*** Ministère Français des Affaires Etrangères (Collège doctoral Franco-Brésilien).

## ABSTRACT-2

**NESTIN EXPRESSION IN THE BRAIN OF RATS SUBMITTED TO *STATUS EPILEPTICUS* INDUCED BY PILOCARPINE: EVALUATION FROM EARLY POST-NATAL DEVELOPMENT TO AGEING**

**Carla Alessandra Scorza^1^, Fulvio Alexandre Scorza^1^, Ricardo Mario Arida^2^, Esper Abrão Cavalheiro^1^, Maria da Graça Naffah-Mazzacoratti^1^**

^1^Neurologia Experimental, Escola Paulista de Medicina Universidade Federal de São Paulo. ^2^Fisiologia, Escola Paulista de Medicina Universidade Federal de São Paulo. E-mail: carlascorza.nexp@epm.br

### Objectives:

Nestin is an intermediate filament protein and an indicator of cellular immaturity. In the adult differentiated cell nestin is replaced for other protein. However, nestin re-expression can be induced in degenerative/ regenerative conditions. In order to understanding plastic alterations after *status epilepticus* (SE) induced by pilocarpine in rats, the present work purposes were: ia) Evaluate physiological hippocampal and cortical nestin expression (P9 to P90); ib) Evaluate hippocampal and cortical alterations of nestin expression in pups submitted to SE (P7-9) and studied from P9-90; ii) Evaluate hippocampal temporal and spatial nestin expression in the acute, silent and chronic phases of the model, iii) Evaluate the hippocampal nestin expression in aged rats and aged rats with epilepsy.

### Methods:

SE induced by pilocarpine (ip) and nestin expression studied by immunohistochemistry.

### Results:

Nestin expression was found in cortical radial glia, hippocampal fibers and blood vessels during early postnatal development, disappearing in adulthood. Rats submitted to SE (P7-9) showed delayed nestin down-regulation. Contrary to normal physiological conditions, we found cortical radial glia in experimental animals of P21 group. Nestin was not detected in adult rats neither in rats of the acute group (SE). However, animals of the silent group presented intense nestin expression at 3 and 7 days after SE, which completely disappeared 14 days after SE. In the chronic phase, the reactive astrocytes expressing nestin were again observed. The aged rats of the control group did not present nestin but aged chronic rats showed the same pattern of nestin immunoreactivity as observed in the chronic adult rats. This work described physiological nestin expression during early postnatal development to adulthood. Nestin down-regulation was delayed in the rats submitted to SE (P7-9), suggesting normal development impairment. In the pilocarpine model, animals of the silent phase presented transient nestin expression in the reactive astrocytes and this protein was detected again in the chronic phase.

### Conclusions:

These data suggests that nestin plays a role in the plastic events after adult brain damage. The normal aging did not induce nestin re-expression. However, aged rats have the potential to re-express this developmental protein. The knowledge of a temporal window of multipotential proteins such as nestin, allows advances in neurobiology understanding. Nestin expression is also correlated with mental disorders and future research could provide better understanding of glia functioning establishing its role as a target for novel neurological and mental disorders treatment, such as antidepressants.

## ABSTRACT-3

**GENETIC RESEARCH IN PSYCHIATRY: PHENOTYPIC REFINEMENT AND ENDOPHENOTYPIC IDENTIFICATION**

**Andréa Poyastro Pinheiro^1,2^, Josué Bacaltchuk^2^**

^1^Department of Psychiatry, University of North Carolina at Chapel Hill, US. ^2^Departamento de Psiquiatria, Escola Paulista de Medicina, Universidade Federal de São Paulo, Brazil. E-mail: apoyastro@ gmail.com or andrea_pinheiro@med.unc.edu

### Objective:

To examine genetic and phenotypic heterogeneity in psychiatric genetics research, and its implications for psychiatric nosology and genetic analyses.

### Methods:

A review of genetic studies in eating disorders, a phenotypic investigation of patterns of menstrual disturbance in eating disorders, and an association analysis of the AKT1 gene and neurocognition in schizophrenia were performed*.

### Results:

1. Genetics in eating disorders^*1^: research findings suggest a substantial influence of genetic factors on the liability to anorexia and bulimia nervosa. Genetic research with admixed populations should take into consideration sample size, density of genotyping and population stratification. The development of a major collaborative initiative in Brazil and South America represents the possibility of studying the genetics of eating disorders in the context of inter ethnic groups, and integrate a new perspective on the biological etiology of eating disorders. 2. Patterns of menstrual disturbance in eating disorders^*2^: eating, weight, and psychopathological traits across menstrual groups of 1.705 women were compared.Menstrual dysfunction occurred across all eating disorder subtypes. Individuals with normal menstrual history and primary amenorrhea reported the highest and lowest lifetime BMI, respectively. Normal menstruation and oligomenorrhea groups reported greater binge eating, vomiting, and appetite suppressant use. Amenorrhea was associated with lower caloric intake and higher exercise. No differences in comorbid Axis I and II disorders were observed. Menstrual dysfunction is not limited to any eating disorder subtype. Menstrual irregularity is an associated feature of all eating disorders. 3. Association of AKT1 & neurocognition in schizophrenia^*3^: Five SNPs (single nucleotide polymorphisms: rs2494732, rs2498799, rs3730358, rs1130241, and rs3803300) were genotyped in 641 individuals with schizophrenia who had participated in the CATIE (Clinical Antipsychotic Trials of Intervention Effectiveness) project. The primary dependent variable was a neurocognitive composite score and exploratory analyses investigated five domain scores (processing speed, reasoning, verbal memory, working memory, and vigilance).

### Results:

There were no significant asymptotic or empirical associations between any SNP and the neurocognitive composite score. An association was observed for the neurocognitive composite score with one of the 5-SNP haplotypes (global score statistic 19.51, df=9, permutation p value=0.02). Exploratory analyses of five domain scores were non-significant for all five SNPs. *AKT1* markers studied are not associated with neurocognition in schizophrenia, and do not support unassessed variation in neurocognitive scores as a reason for this discrepancy.

### Conclusions:

Improvement in phenotypic definitions of psychiatric disorders and the development of endophenotypes that index disease both genetically and phenotypically may allow more refined genetic analyses.

## ABSTRACT-4

**PARENTHOOD IN SCHIZOPHRENIA: THE IMPACT OF THE DISEASE IN PATIENTS AND THEIR CHILDREN’S LIVES**

*****Angela Cristina Cesar Terzian^1^, Sérgio Baxter Andreoli^2^, John McGrath^3^, Jair de Jesus Mari^4^*****

^1^Assistant Professor, Department of Clinical Medicine, Universidade Federal do Mato Grosso (UFMT). ^2^Affiliated Professor, Department of Psychiatry, Universidade Federal de São Paulo (UNIFESP). ^3^Professor, Department of Psychiatry, University of Queensland, Queensland Centre for Mental Health Research, Australia. ^4^Professor, Department of Psychiatry, Universidade Federal de São Paulo (UNIFESP). E-mail: terzian.angela@gmail.com

### Objectives:

To determine the reproduction rates of patients with schizophrenia and comparing findings with population rates. To identify the social adjustment of the adult’s offspring’s of these patients.

### Methods:

Patients diagnosed with schizophrenia attending the public mental health clinics in the city of Cuiaba (Mato Grosso State) answered a standardized questionnaire. Three outcomes were investigated: marital rate (proportion of subjects ever married), fertility (ability to procreate), and fecundity (number of offspring produced by a fertile individual). In a second step, patients and another healthy member of the family provided information about their children by way of a structured questionnaire. Data from the Brazilian census was used for comparing population rates.

### Results:

489 patients with diagnosis of schizophrenia were identified; 232 (47.4%) were men. Mean current age was 41.5 years (SD=11.6). The marital rate was 42.2% for male patients and 74.7% for female patients. Ninety-two (39.7%) of the male patients were fathers and 202 (78.6%) of the female patients had at least one live-born infant. These differences were statistically significant (p<0.001). Fecundity was 3.2 for men and 3.4 for women (p=0.57). Male patients were significantly less frequently married than same-age males in the general population. There was a difference statistically significant only in fertility rate for women in the age range 45 years and over (female patients: 83.8% (CI^95%^ 76.8–90.8); female population: 92.3%). There were no differences statistically significant regarding marital rate, fertility for women in the range 25-44 years, and fecundity. 294 patients reported being a parent and a total of 828 children were identified; 431 were 18 years or older and were thus included in the study. The percentage of age-grade discrepancy was 59.2% (CI^95%^ 45.4–73.0) for the patients’ offspring at the ages of 18 and 19 years, and of 71.1% in the general population, a difference not statistically significant. The patients’ offspring had lower employment situation when compared with the general population (66.7% and 75.6% respectively; CI^95%^ 62.1–71.3). Sons were less married than the general male population (54.7% and 66.0% respectively; CI^95%^ 48.2–61.2).

### Conclusions:

Adult offspring of patients with schizophrenia had social adjustment problems that were markedly reflected in employment and marital status. Our results, obtained from the evaluation of every day life variables and population comparisons, showed that the offspring of patients with schizophrenia have social adjustment impairments in their adult lives, particularly in terms of employment, and, therefore, should be identified as a vulnerable group. For these individuals, early preventive strategies should be developed to minimize the unfavorable impact of having parents with schizophrenia.

***Financial support:*** CNPq Grant 474400/03-4. Angela Terzian was financed through a scholarship from Capes. Jair Mari is an I-A researcher with the CNPq.

## ABSTRACT-5

**BRAIN CIRCUITS INVOLVED IN MANIFESTATIONS OF IRRITABILITY IN HEALTHY HUMANS: A STUDY USING FUNCTIONAL MAGNETIC RESONANCE IMAGING**

**Carlos T. Cerqueira^1^, Jorge R.C. Almeida^1^, Clarice Gorenstein^1^, Valentim Gentil Filho^1^, Claudia C. Leite^2^, Edson Amaro Jr^2^, Geraldo F. Busatto^1,2^**

^1^Departments of Psychiatry and ^2^Radiology, University of São Paulo School of Medicine, Brazil. E-mail: carlostclabneuropq@hotmail.com

Irritability is a negative emotional state distinguishable from anger, which may emerge either in healthy subjects or in the context of major depressive, bipolar or other psychiatric disorders. Objective: To investigate the brain circuitry underlying personal script-driven irritability in healthy subjects (n=11) using functional magnetic resonance imaging (fMRI).

### Methods:

Blood-oxygen level dependent (BOLD) changes were recorded with fMRI during auditory presentation of personal scripts of irritability, happiness or neutral emotional content, over three sequential runs. Self-rating emotional measurements and skin conductance recordings were obtained during image acquisition.

### Results:

During the irritability condition, self-rating scores for irritability and sadness were significantly greater relative to the two other conditions. Compared to neutral scripts, increased BOLD signal during irritability scripts was detected in the left subgenual cingulate cortex, and in medial, antero-lateral and postero-lateral portions of the dorsal prefrontal cortex. The same regions, except the medial and postero-lateral dorsal prefrontal cortex, and the temporo-occipital region additionally, showed significantly increased BOLD signal during irritability scripts relative to the happiness condition. The happiness condition, when compared to the neutral condition, showed increased BOLD signal again in the medial frontal gyrus and the postero-lateral portion of the dorsal prefrontal cortex, as well as in regions not activated during the irritability state, including the left anterior insula, thalamus and hypothalamus, as well as the middle temporal gyrus bilaterally. There was a trend towards greater skin conductance levels associated with the happiness condition relative to the irritability condition in the first run, and this difference was progressively attenuated during the subsequent runs.

### Conclusion:

The findings related to the irritability condition partially overlap with the results of previous functional imaging studies of normal sadness induction and major depression, and differ from those reported during anger induction. The activation of the subgenual cingulate gyrus and antero-lateral prefrontal regions was specific to the irritability condition, and may relate to the known prominence of cognitive processing aspects during irritability states. The activation of medial and posterior dorsolateral portions of the prefrontal cortex during both the irritability and happiness conditions is consistent with the previous literature, and may relate to general aspects of emotional processing.

## ABSTRACT-6

**PROTON SPECTROSCOPY STUDY OF THE DORSOLATERAL PREFRONTAL CORTEX IN PEDIATRIC DEPRESSED PATIENTS**

**Sheila Cavalcante Caetano, Beny Lafer**

Departments of Psychiatry, University of São Paulo School of Medicine, Brazil. E-mail: scaetano_2000@yahoo.com

Magnetic resonance imaging (MRI) and proton magnetic resonance spectroscopy (^1^H MRS) have been applied to anatomical and neurochemical studies of adult patients with Major Depressive Disorder (MDD). These have demonstrated abnormalities in specific structures that are involved in mood regulation, expression and recognition.

### Objectives:

To investigate the hypotheses that children and adolescents with MDD would present both smaller left dorsolateral prefrontal cortex (DLPFC) and left hippocampal volumes compared to healthy children; and no significant abnormalities in amygdala, caudate, putamen, thalamus, anterior cingulate and total brain volumes; and lower levels of glycerophosphocholine plus phosphocholine (GPC+PC; or choline-containing-compounds) and higher myo-inositol levels in the left DLPFC.

### Methods:

Nineteen children and adolescents (9 off medication), mean age of 13.0 (±2.4) years old, who fulfilled diagnostic criteria for MDD (DSM-IV) and 24 healthy controls, mean age of 13.9 (±2.9) years old, were evaluated on a 1.5 Tesla (Philips Intera 8.1.1.) MRI scanner to obtain volumetric measurements. These children and adolescents were evaluated at the Department of Psychiatry at the University of Texas Health Science Center ar San Antonio. We also conducted single voxel of the left DLPFC in 14 (73.7%) of the 19 patients with MDD, mean age of 13.3±2.3 years old, and in 22 (91.7%) of the 24 healthy controls, mean age of 13.6 (±2.8) years old.

### Results:

Compared to healthy controls, children and adolescents with MDD presented: (i) significantly smaller left hippocampal gray matter volumes (p=0.032), and (ii) significantly lower levels of glycerophosphocholine plus

phosphocholine [GPC+PC; or choline-containing-compounds (p=0.002)] and higher myo-inositol levels [Ino (p=0.001)] in the left DLPFC.

### Conclusions:

Our findings of smaller left hippocampal volumes in children and adolescents with MDD are in agreement with studies conducted in adults with MDD. Lower levels of choline-containing-compounds (GPC+ PC) in pediatric patients with MDD may reflect lower cell membrane turn-over. Higher myo-inositol levels in MDD may represent a disturbed secondary messengers system. Our findings provide further support to the existence of anatomical and neurochemical abnormalities in children and adolescents with MDD.

## ABSTRACT-7

**DOES RESTRICTING OPENING HOURS REDUCE ALCOHOL RELATED VIOLENCE?**

***Sergio Duailibi PhD^1^, William Ponicki MA^2^, Joel Grube PhD^2^, Ilana Pinsky PhD^1^, Ronaldo Laranjeira PhD^1^, Martin Raw PhD^3^***

^1^Unidade de Pesquisa em Álcool e outras Drogas (UNIAD); Departamento de Psiquiatria, Universidade Federal de São Paulo, Sao Paulo, Brazil. ^2^Prevention Research Center, Berkeley, California, USA. ^3^Department of Health Policy, University of Nottingham, England. E-mail: duailibi@uol.com.br

### Objective:

To investigate the effect of limiting the hours of sale of alcoholic drinks on violence against women and homicides in the Brazilian city of Diadema. The policy, introduced in July 2002, prohibited on-premises alcohol sales after 11pm.

#### Methods:

Data on homicides (1995 to 2005) and violence against women (2000 to 2005) from the Diadema (population 360,000) police archives were analyzed using loglinear regression analyses and other models. All models included controls for percentage monthly unemployment rates, interaction between local economic conditions, crime rates, and policy factors affecting violence. Data limitations restricted our ability to control for changes over time in local characteristics such as age distribution, poverty rate, number of alcohol outlets.

#### Results:

The introduction of the new restriction on drinking hours led to a decrease of almost 9 murders a month. Assaults against women also decreased but this impact was not significant in models that controlled for underlying trends. The model with linear time trends estimates an insignificant reduction of 225 assaults (a 21% drop from predicted assaults without the intervention), with a 95% confidence interval of 98 more to 548. All models indicated that a significant reduction in homicides. The preferred model, controlling for both prior enforcement changes and linear time trends, suggests that 319 homicides were prevented during the first three years of the new law, a 44% decline from what would be expected without the law. This estimate has a 95% confidence interval ranging from 193 to 445 fewer homicides over this period.

#### Conclusion:

Our analyses suggest that closing the bars at 11pm produced a large and statistically significant reduction in homicides – almost 9 murders a month in a city of 360,000 residents – an annual reduction of 106 or 30 per 100,000 population. This is a considerable public health achievement, especially in a country with such a high level of violent deaths. Our assaults findings are weaker than our homicide results possibly because of the shorter time series of available data. Thus although the data are consistent with a sizeable reduction in assaults against women we are less certain that this effect is due to the new law. Introducing restrictions on opening hours resulted in a significant decrease in murders, confirming what we know from the literature, that restricting access to alcohol can reduce alcohol related problems. These results give no support to the converse view, that increasing availability will somehow reduce problems.

## ABSTRACT-8

**CONTINUITY OF BEHAVIOURAL AND EMOTIONAL PROBLEMS FROM PRE-SCHOOL YEARS TO INITIAL ADOLESCENCE: A BIRTH COHORT STUDY**

**Luciana Anselmi^1^, Fernando C. Barros^2^, Maycoln Theodore^3^, César A. Piccinini^4^, Thomas M. Achenbach^5^, Luis A. Rohde^6^**

^1^Post-Graduate Program in Psychiatry, Federal University of Rio Grande do Sul, and Post-Graduate Program in Epidemiology, Federal University of Pelotas, Brazil; ^2^Pan-American Health, Organization/World Health Organization, Latin American Center for Perinatology, Uruguay; ^3^Post-Graduate Program in Psychology, Unisinos, Brazil; ^4^Institute of Psychology, Federal University of Rio Grande do Sul, Brazil; ^5^Research Center for Children, Youth, and Families, University of Vermont, USA; ^6^Child and Adolescent Psychiatric Division, Federal University of Rio Grande do Sul, Brazil. E-mail: luanselmi@terra.com.br

All previous longitudinal community studies assessing the continuity of child behavioral/emotional problems were conducted in developed countries.

### Objective:

To evaluate an 8-year follow-up of behavioral/emotional problems firstly assessed during pre-school years in a developing country.

### Methods:

601 children randomly selected from a Brazilian birth cohort were evaluated by their parents at 4 and 12 years through the same standard procedure – Child Behavior Checklist (CBCL).

### Results:

Behavioral/emotional problems presented a moderate stability (r=.42). Externalization problems presented higher stability and a more homotipic continuity than internalization problems. There was a decrease of about 4 points in the CBCL Total Problems mean scores from 1997 to 2005. From the children presenting deviating scores at the age of 4, 31% remained deviating at the age of 12, but the disorder presented has changed with age. Most children (70%) with an appropriate functioning at 4 years old remained well adapted at 12 years old, while only 4.7% of them became deviant. The Total Problems score at 12 years old was predicted by Rule-Breaking Behavior [OR=7.29, 95% CI 2.71–19.57] and Social Problems [OR=3.95, 95% CI 1.38–9.32] scores at 4 year old. Externalizing syndromes (Rule-Breaking or Aggressive Behavior) were isolated or part of the predictors for the three general CBCL scores and six out of the eight CBCL syndromes.

### Conclusions:

Behavioral/emotional problems in preschool children persist moderately to the onset of adolescence in community samples. The results are very similar to other international studies and similar changes in the characteristics of different cohorts seem to show a developmental effect of age in the evolution of the behavioral/emotional problems. It seems that aggressive or rule-breaking behavior at the age of 4 are a risk factor for different outcomes in mental health comprising the developmental history of most behavioral/emotional problems at the onset of adolescence. It is possible to suppose that several kinds of psychopathologies appear as externalizing problems at preschool age because small children are more likely to use actions to express conflicts, anxieties or even thoughts in a developmental stage during which the most complex psychic mechanisms involving symbolization, communication of feelings and drive restraint remain restrict. Likewise, the internalizing problems that take place during childhood are expressed in several different ways at this age due to the lack of cognitive structures that make it possible for the individual to experience feelings of guilt or uselessness. Therefore, externalizing problems are strong disorder predictors at pre-school age.

## ABSTRACT-9

**ASSOCIATION BETWEEN ENVIRONMENTAL AND GENETIC FACTORS AND ATTENTION DEFICIT/HYPERACTIVITY DISORDER – PREDOMINANTLY INATTENTIVE SUBTYPE: SMOKING DURING PREGNANCY AND ADRENERGIC AND DOPAMINERGIC CANDIDATE GENES**

***Marcelo Schmitz^1^, Daniel Denardin^1^, Tatiana Laufer da Silva^1^, Thiago Pianca^1^, Mara Helena Hutz^1^, Luis Augusto Rohde^1^, Tatiana Roman^2^, Stephen Faraone^3^***

^1^Universidade Federal do Rio Grande do Sul. ^2^Fundação Faculdade Federal de Ciências Médicas de Porto Alegre. ^3^SUNY Upstate Medical University, New York. E-mail: mschmitz@ orion.ufrgs.br

### Objective:

To investigate the association between attention deficit/hyperactivity disorder – predominantly inattentive subtype (ADHD-I) and prenatal exposure to nicotine and four candidate genes (α-2A adrenergic receptor [ADRA2A], dopamine D4 receptor [DRD4], dopamine transporter [DAT1], and dopamine beta-hydroxylase [DBH]).

### Methods:

In family-based and case-control association studies, we assessed 100 children and adolescents (and their parents) with ADHD-I and 100 non-ADHD controls. Cases and controls were matched by gender and age. Subjects were screened using teacher reports in the SNAPIV rating scale in 12 public schools of Porto Alegre, Brazil. Diagnosis of ADHD-I and co-morbidities were performed according to DSM-IV criteria using semi-structured (K-SADS-E) + clinical interviews with both the subjects and their parents. Prenatal exposure to nicotine and demographics were evaluated by direct interview with biological mothers. Potential confounders were defined based on conceptual analyses of the literature and/or using a broad statistical definition (association with both the study factor and outcome for a p≤0.20).

### Results:

In univariate analyses, a significant association between smoking during pregnancy (defined both categorically and dimensionally) and ADHD-I was found. After adjusting for confounders, children whose mothers smoked ≥10 cigarettes during pregnancy presented an odds ratio significantly higher for ADHD-I than children who were not exposed to nicotine during pregnancy (OR=3.44; CI_95_=1.17-10.06). The dimensional analysis showed significantly higher inattention scores in subjects exposed to nicotine during the intrauterine period than in controls after adjusting for confounders (p=0.002). The haplotype relative risk (HRR) analysis and the transmission disequilibrium tests (TDTs) showed no significant associations between these candidate genes and ADHD-I. In the case-control approach, we verified that the homozygosis of the G allele for the *ADRA2A* was significantly more frequent in the ADHD-I probands (20%) than in controls (8%), even after adjusting for potential confounders (OR=3.78; CI_95_=1.23-11.62).

### Conclusions:

ADHD-I seems to be associated with prenatal exposure to nicotine. In a non-clinical sample, we obtained results that concur with previous findings of other studies involving subjects with general ADHD. Our results on candidate genes suggest that the *ADRA2A* is associated with ADHD-I, replicating findings from other studies in the literature on the importance of the noradrenergic system in the pathophysiology of ADHD, especially for the dimension of inattention.

## ABSTRACT-10

**PATTERNS OF CEREBRAL FUNCTIONING DURING LANGUAGE PRODUCTION: FUNCTIONAL MAGNETIC RESONANCE IMAGING STUDIES USING VERBAL FLUENCY TASKS WITH STIMULI OF DIFFERENT LEVELS OF DIFFICULTY**

Maurien Senhorini^1*^, Edson Amaro Jr^2^, Jorge R.C. Almeida^1^, Carlos T. Cerqueira^1^, Maristela S Schaufelberger^1^, Claudio Campi de Castro^2^, Geraldo F. Busatto^1,2^

^1^Department of Psychiatry, Faculdade de Medicina da Universidade de São Paulo (USP). ^2^Department of Radiology, Faculdade de Medicina da Universidade de São Paulo (USP). ^*^Dissertação, Departamento de Psiquiatria, Faculdade de Medicina da Universidade de São Paulo, 2006.

The neural circuits involved during language production can nowadays be mapped using functional Magnetic Resonance Imaging (fMRI) during performance of neuropsychological tasks, most often using phonological verbal fluency paradigms. These studies have demonstrated the engagement of multifocal brain circuits using such tests, with emphasis on the involvement of portions of the prefrontal and anterior cingulate cortices. In English, phonological fluency tests usually employ, as stimuli, the letters F-A-S, which are considered easy. However, the level of difficulty to produce words from these and other letters in Portuguese is not known. Moreover, there are few neuroimaging studies in the international literature evaluating whether the use of F-A-S or other letters of greater difficulty leads to distinct patterns of brain activity.

### Objectives:

This study aimed to evaluate the patterns of brain functioning in healthy subjects during verbal fluency performance using letters of varying levels of difficulty to produce words.

### Methods:

Nine healthy subjects were assessed with fMRI during a phonological verbal fluency task carried out using words beginning with easy or moderately difficult letters in Portuguese. Letters were selected with basis on a pilot study with 74 healthy volunteers who performed a verbal fluency test in its traditional format, using F-A-S and 14 other letters.

### Results:

In the off-scanner study, we demonstrated differences in the level of difficulty between the letters studied, both in terms of the number of words articulated and the latency of time to produce them (Senhorini et al. J Clin Exp Neuropsychol 28:1191-200, 2006). In the fMRI experiment, there was greater activation of the anterior cingulate cortex during the production of words beginning with the moderately difficult letters; besides, there was activation of the prefrontal cortex during both tasks, restricted to the left hemisphere when easy letters were used, and more extensively and bilaterally during the production of words with moderately difficult letters.

### Conclusions:

Our fMRI results confirm the notion that the level of difficulty to perform phonological verbal fluency tests determines the greater engagement of anterior cortical areas known to be relevant to verbal production.

## ABSTRACT-11

**PERITRAUMATIC TONIC IMMOBILITY PREDICTS A POOR RESPONSE TO PHARMACOLOGICAL TREATMENT IN VICTIMS OF URBAN VIOLENCE WITH PTSD**

**Adriana Fiszman^1^, Carla Marques-Portella^1,2^, Mauro V. Mendlowicz^1,3^, Eliane Volchan^2^, Evandro S.F. Coutinho^4^, Wanderson F. Souza^4^, Vanessa Rocha^2^, Alessandra A. Lima^1^, Fernanda P. Salomão^1^, Jair J. Mari^5^, Ivan Figueira^1^**

^1^Institute of Psychiatry, Universidade Federal do Rio de Janeiro (IPUB- UFRJ). ^2^Institute of Biophysics Carlos Chagas Filho, Universidade Federal do Rio de Janeiro (IBCCF-UFRJ). ^3^Department of Psychiatry and Mental Health, Universidade Federal Fluminense (MSM-UFF). ^4^Department of Epidemiology, Escola Nacional de Saúde Pública (ENSP – FIOCRUZ). ^5^Department of Psychiatry and Medical Psychology of the São Paulo Medical School, Universidade Federal de São Paulo (EPM-UNIFESP). E-mail: adrianafiszman@terra.com.br

Tonic immobility is the ultimate resort in antipredator behavior when fight/flight responses result ineffective as the prey founds itself physically restrained and cannot escape. It is characterized by paralysis with rigidity and analgesia. To date, tonic immobility was systematically assessed in humans by only two studies dealing with women victims of sexual-related trauma.

### Objectives:

This study evaluated the prevalence of peri-traumatic tonic immobility (PTI) in patients with post-traumatic stress disorder (PTSD) and probed the correlation between PTI and the response to pharmacological treatment of post-traumatic stress symptoms.

### Methods:

Victims of urban violence with PTSD diagnosed through the Structured Clinical Interview for DSM-IV Axis I Disorders (n=23) underwent a naturalistic pharmacological treatment according to the recommended guidelines for PTSD. The Post-Traumatic Stress Disorder Checklist - Civilian Version (PCL-C) and the Clinical Global Impressions (CGI) Severity scores were applied at baseline and endpoint. PTI was assessed using the Tonic Immobility Scale. PCL-C and CGI scores data at baseline and endpoint were compared among patients with and without PTI.

### Results:

PTI was reported by 43% of the patients, with men showing a higher prevalence than women (8/14 and 2/9, respectively). The group without PTI responded better to treatment: their average PCL-C and CGI scores dropped significantly more in comparison to the group with PTI (p<.05 and p<.001, respectively). Further, at the endpoint, only 1 out of 10 patients with PTI presented their PCL-C scores below the cut-off for PTSD (50), as compared to 7 out of 13 patients without PTI. The two groups were not significantly different in terms of treatment length.

### Conclusions:

This study has expanded the scope of two previous investigations on PTI in women sexually abused by showing its occurrence also in men and during nonsexual violence. As predicted, a significant relationship between the occurrence of PTI and poor response to the standard pharmacological treatment for PTSD was observed, thus suggesting that PTI may carry a prognostic value in this disorder. Although the biological underpinnings of tonic immobility are yet to be unveiled, clinical and experimental evidence indicates that they possibly involve analgesia-related, opioid-dependent mechanisms. Additional investigations with a prospective design and involving a large and representative cohort are needed to expand the knowledge in this promising area and to encourage the adoption of innovative diagnostic and therapeutic approaches.

## ABSTRACT-12

**PUSHER BEHAVIOR IN A TERTIARY UNIVERSITY HOSPITAL: INCIDENCE, PROSPECTIVE FUNCTIONAL EVALUATION AND ITS CORRELATION WITH NEUROIMAGING DATA**

***Santos-Pontelli TEG, João Pereira Leite***

Faculdade de Medicina de Ribeirão Preto, Universidade de São Paulo, Ribeirão Preto.

Pusher behavior (PB) is an intriguing disorder of postural control that may affect hemiparetic patients with hemispheric lesions. Patients with the PB extend the unaffected arm, actively push themselves away toward the paretic side and resist any attempt of passive correction toward the earth-vertical upright orientation as a result of a severe conflict between the perception of body and visual verticality. Although the first descriptions of the PB date back more than 20 years, there are several aspects of it that remain unclear.

### Objectives:

To investigate the occurrence and clinical features of PB in patients with acute encephalic lesions; to assess the prognosis of the PB; to identify the brain areas related with the PB; to evaluate horizontal semicircular canal function in patients with PB.

### Methods:

Prospectively, we screened the pusher inpatients of the Neurology Impatient Ward of the HCFMRP/USP Emergency Unit during a 3.5 years period. Patients were evaluated using a standardized Scale for Contraversive Pushing (SCP), neurological and neuropsychological examination, activities of daily living function, and neuroimaging workup. To analyze the semicircular canals function, we applied caloric and rotatory tests in 9 patients with stroke and PB.

### Results:

We found 31 patients with severe PB, mean age 67.4±11.89, 25 patients with stroke, 5 with cranial trauma (CT) and 1 with cranial hemorrhagic metastasis. Hemianopia was observed in 64.5% of pusher patients, hypoesthesia in 61.3%, dysphagia in 100%, hemineglect in 35.5% and anosognosia only in 2 of the evaluated patients. The median recovery time of the PB was 53 days (8-789 days). Compared with stroke patients, the severity of PB and the recovery time of the patients with CT were significantly shorter. The middle cerebral artery was found the most affected topography in ischemic stroke pusher patients. The thalamus and posterior parietal lesions were more frequently observed in pusher than control patients. The midline shift and the hemorrhagic stroke volume did not influence the severity of PB or its prognosis. On vestibular function tests, neither the directional preponderance nor the canal paresis correlated with the pushing severity or its prognosis.

### Conclusions:

1) the relative frequency of the PB in our hospital is 1.5%. 2) the PB occurs in non-stroke patients; 3) the recovery time of the PB can overtake two years; 4) the pusher patients with CT exhibit less severe PB and better prognosis than stroke pusher patients; 5) age, previous encephalic lesion, hypoesthesia, hemianopia and anosognosia are not fundamental for the PB outcome; 6) the severity of the pusher symptoms influences its prognosis; 7) the midline shift and the intracerebral hemorrhage volume do not correlate with the severity or the recovery time of pusher symptoms; 8) a dysfunction of semicircular canals does not seem to be relevant for the clinical manifestations of the PB.

***Financial support:*** Fundação de Apoio a Pesquisa do Estado de São Paulo (FAPESP), Conselho Nacional de Desenvolvimento Científico e Tecnológico (CNPq), Fundação de Apoio ao Ensino, Pesquisa e Assistência do Hospital das Clínicas da Faculdade de Medicina de Ribeirão Preto da Universidade de São Paulo (FAEPA).

